# CT radiomic models to distinguish COVID-19 pneumonia from other interstitial pneumonias

**DOI:** 10.1007/s11547-021-01370-8

**Published:** 2021-05-27

**Authors:** Nicolò Cardobi, Giulio Benetti, Giuseppe Cardano, Cinzia Arena, Claudio Micheletto, Carlo Cavedon, Stefania Montemezzi

**Affiliations:** 1 Department of Pathology and Diagnostics, Radiology Unit, Azienda Ospedaliera Universitaria Integrata, P.le Stefani 1, 37126 Verona, Italy; 2 Department of Pathology and Diagnostics, Medical Physics Unit, Azienda Ospedaliera Universitaria Integrata, P.le Stefani 1, 37126 Verona, Italy; 3 Cardiovascular and Thoracic Department, Pneumology Unit, Azienda Ospedaliera Universitaria Integrata, P.le Stefani 1, 37126 Verona, Italy; 4 Università di Verona, Verona, Italy

**Keywords:** COVID-19, Lung, Pneumonia, Viral, Machine Learning, Tomography, X-Ray Computed

## Abstract

**Purpose:**

To classify COVID-19, COVID-19-like and non-COVID-19 interstitial pneumonia using lung CT radiomic features.

**Material and Methods:**

CT data of 115 patients with respiratory symptoms suspected for COVID-19 disease were retrospectively analyzed. Based on the results of nasopharyngeal swab, patients were divided into two main groups, COVID-19 positive (C +) and COVID-19 negative (C−), respectively. C− patients, however, presented with interstitial lung involvement. A subgroup of C−, COVID-19-like (CL), were considered as highly suggestive of COVID pneumonia at CT. Radiomic features were extracted from the whole lungs. A dual machine learning (ML) model approach was used. The first one excluded CL patients from the training set, eventually included on the test set. The second model included the CL patients also in the training set.

**Results:**

The first model classified C + and C− pneumonias with AUC of 0.83. CL median response (0.80) was more similar to C + (0.92) compared to C− (0.17). Radiomic footprints of CL were similar to the C + ones (possibly false negative swab test). The second model, however, merging C + with CL patients in the training set, showed a slight decrease in classification performance (AUC = 0.81).

**Conclusion:**

Whole lung ML models based on radiomics can classify C + and C− interstitial pneumonia. This may help in the correct management of patients with clinical and radiological stigmata of COVID-19, however presenting with a negative swab test. CL pneumonia was similar to C + pneumonia, albeit with slightly different radiomic footprints.

## Introduction

Initial reports of coronavirus disease 2019 (COVID-19) were in China in December 2019 [1]. After few months, on March 11, 2020, the World Health Organization announced COVID-19 outbreak as pandemic. The lung is the most affected organ, although the involvement of different organs has been demonstrated [2, 3].

The most used confirmatory test of this infection is the isolation of the viral genome, by means of reverse transcriptase polymerase chain reaction (RT-PCR), on upper airways specimen usually obtained by nasopharyngeal swab. Albeit a rather simple and non-invasive method to prove the infection, it is affected by a false negative rate up to 30% [4, 5].

Diagnostic imaging occupies a pivotal role, with two diagnostic modalities: chest radiography and high-resolution computed tomography (HRCT). The former is of rapid execution, easily available and characterized by a low dose. On the other hand, it may not be able to demonstrate the sometimes nuanced changes induced by the infection, especially in the early stages of the disease [6]. On the contrary, HRCT has proven to be a very sensitive technique in finding these alterations, but it is relatively unspecific. HRCT signs of COVID-19 parenchymal pulmonary infection, mainly represented by multiple and subpleural ground-glass (GGO) parenchymal opacities, can be superimposed on those of a generic interstitial viral pneumonia, hence the low specificity of the method [7].

The extraction of quantitative parameters from images is becoming increasingly important in Radiology. Some of these parameters fall within the general concept of Radiomics, i.e., the analysis of medical images aimed at obtaining quantitative information that cannot be detected through their simple visual observation [8, 9]. Radiomics has been applied with excellent results to various radiological fields, mainly in oncology, often reaching a significant prognostic value [10–13]. It has been used in the distinction between inflammatory/infectious and oncological disease [14], in the discrimination between primary tuberculous pneumonia and acquired pneumonia in children [15] and in the prediction of the onset of immunotherapy-induced pneumonia [16]. This approach has recently been used, as regards SARS-CoV-2 pneumonia, in predicting the duration of hospitalization in affected patients [17] and in a preliminary study regarding the distinction between COVID-19 and non-COVID-19 pneumonia [18].

This study aims to evaluate whether radiomics is useful in distinguishing COVID-19 pneumonia from non-COVID-19 interstitial pneumonia, thus alleviating the problem of partial overlap of qualitative findings in the two groups. The classification of the two forms would allow a better management of the patient even if a diagnosis of positivity from COVID-19 is not yet available, due to processing delay of the swab test or when the latter resulted in a false negative.

## Materials and methods

### Study cohort

A total of 115 patients who performed HRCT for respiratory symptoms suspected for COVID-19 disease were selected. All patients had a swab test, resulting in 68 COVID-19 positive (C +) and 47 COVID-19 negative (C−) cases. The latter are patients admitted to the emergency department with COVID-19 like respiratory symptoms (fever, cough, dyspnea) but with negative COVID-19 swab. Since the negative test, they underwent chest HRCT to rule out the possible presence of COVID-like pneumonia. In this group, we isolated a subgroup of 9 patients, called COVID-like (CL), in which the HRCT findings were highly suggestive of COVID-19 disease.

The HRCT images were reviewed by a radiologist to assess the presence/absence of these findings: ground-glass opacities (GGO, increased lung density without obscuration of the underlying vessels), consolidation (homogeneous increased lung density with obscuration of the vessels), crazy paving (GGO with a reticular thickening of the interlobular septa), subpleural bands, fibrotic irregular stripes, microvascular dilatation and traction bronchiectasis. The reviewer radiologist evaluated these findings and assigned a CO-RADS [19] score to each patient. Mean and median CO-RADS score for C− and CL patients were then calculated.

### CT scan

All HRCT scans were acquired with the same CT scanner (GE Revolution Evo, General Electric Healthcare). Both lung and standard reconstructions were obtained. CT protocol was set as follows: 120 kV, 80 mA (automatic exposure control employed), rotation time of 0.7 s, pitch of 1 mm, and detector collimation of 0.7 mm. The scanning range was from the thorax inlet to the posterior costal angle, with patient keeping breath hold at full inspiration. Lung reconstruction settings were: window width 1600 HU, window level-600 HU, slice thickness 1.25 mm, slice interval 1.25 mm, matrix 512 × 512; adaptive statistical iterative reconstruction (ASIR-V) set to 30% was used. Standard reconstruction settings were: window width 400 HU, window level 40 HU, slice thickness 2.5 mm, slice interval 2.5 mm, matrix 512 × 512, ASIR-V set to 60%.

### Lung segmentation

The DICOM files were converted to nrrd format by using 3D Slicer(v4.10.2) and pre-processed using homemade scripts in Python(v3.7.6) under conda(v4.8.2) environment. Since the radiomic features are intrinsically dependent on voxel size and shape [20], both the STD and LUNG images were resampled to cubic voxels of 0.7 mm through the bi-cubic splines interpolation provided by SimpleITK(v1.2.4). The lungs in both the STD and LUNG reconstructions were automatically contoured though the *lungmask*(v0.2.8) package, by exploiting the U-net(R231) convolutional network trained on COVID patients (R231CovidWeb).

### Radiomic features extraction

Radiomic features were extracted from the resampled CT images by using lung masks and pyradiomics(v3.0), an open-source python package. According to the IBSI [20] guidelines, the CT gray values were not modified or limited and were shifted by 1000 HU to prevent negative values. All the available features implemented in pyradiomics were extracted: Shape, First Oder, GLCM (Gray-level co-occurrence matrix), GLDM (Gray-level dependent matrix), GLRLM (Gray-level run-length matrix), GLSZM (Gray-level size zone matrix) and NGTDM (Neighboring Gray Tone Difference Matrix). These radiomic features were extracted from both the original STD and LUNG reconstructions and from a set of derived volumes: three different Laplacian of Gaussian (LoG) with a sigma of 1, 2 and 3 mm, respectively; Logarithm; Exponential; Gradient; Local Binary Pattern (LBP3D) with the default value of 1 for the radius; Coiflets 1 Wavelet with one level. For the features requiring a gray-level discretization, a fixed bin-width of 25 HU was adopted, similarly to other works where radiomics had been applied to lung CT [21, 22]. A total number of about 1700 features were extracted from both reconstructions.

### Data preparation

Statistical analysis was performed using R(v3.6.3) in RStudio(v1.3.959). Data coming from the STD and LUNG reconstructions were merged, creating a pool of about 3400 features. To reduce the redundancy of information inside the data set, highly correlated variables were removed. Starting from a correlation threshold of 0.99, the absolute pairwise correlation between all the features was estimated using the Pearson’s coefficient. When two features were correlated so that to exceed the threshold, the one with the highest average correlation with all the other features was removed. This process was re-iterated decreasing the threshold to 0.90 in steps of 0.01. After the removal of the redundant features, the number of available covariates to build the model was 681 (80% removed).

### Model building

The first regularized logistic model was trained excluding CL patients. The training set was composed of 80% of the C + and C− patients whereas the test set was composed of the rest of the patients, including CL ones. The covariates of both sets were centered to 0 and scaled to 1 by using the mean and the standard deviation of the training set. Then, the best value for the regularization hyperparameter was computed by repeating 100 times a tenfold cross-validated least absolute shrinkage and selection operator (LASSO) regression model and averaging the lambda values which maximized the AUC. With the optimal lambda value, a LASSO model was trained on the whole training set and used to predict the probabilistic outcome in the test set. The comparison between the predicted probabilities for the three classes (C−, C + and CL) was performed by means of the two-sample Welch t-test. The median predicted value of each class was considered representative of the *class response*. The performance of the classifier was estimated through the ROC curve, in the case of CL exclusion (*i.e.,* only C + and C−) and in the case of CL inclusion as COVID-19 positive patients in the test set.

For the second LASSO model, CL patients were included in both the training (80%) and test (20%) sets. In this case, the small amount of CL patients required a balanced (stratified) split. From the 38/68/9 patients in the C−/C + /CL class, the number of patients lying in the training set was 30/54/7. The following pipeline is identical to the previous model, with CL patients considered C + after the initial split. The performance of the regularized model was estimated by using the ROC curve and the optimal classifier threshold was computed as the one maximizing the sum of sensitivity and specificity (equivalent to maximizing the Youden’s index). All the ROC curves were computed through the pROC package(v1.16.2).

**Fig. 1:**
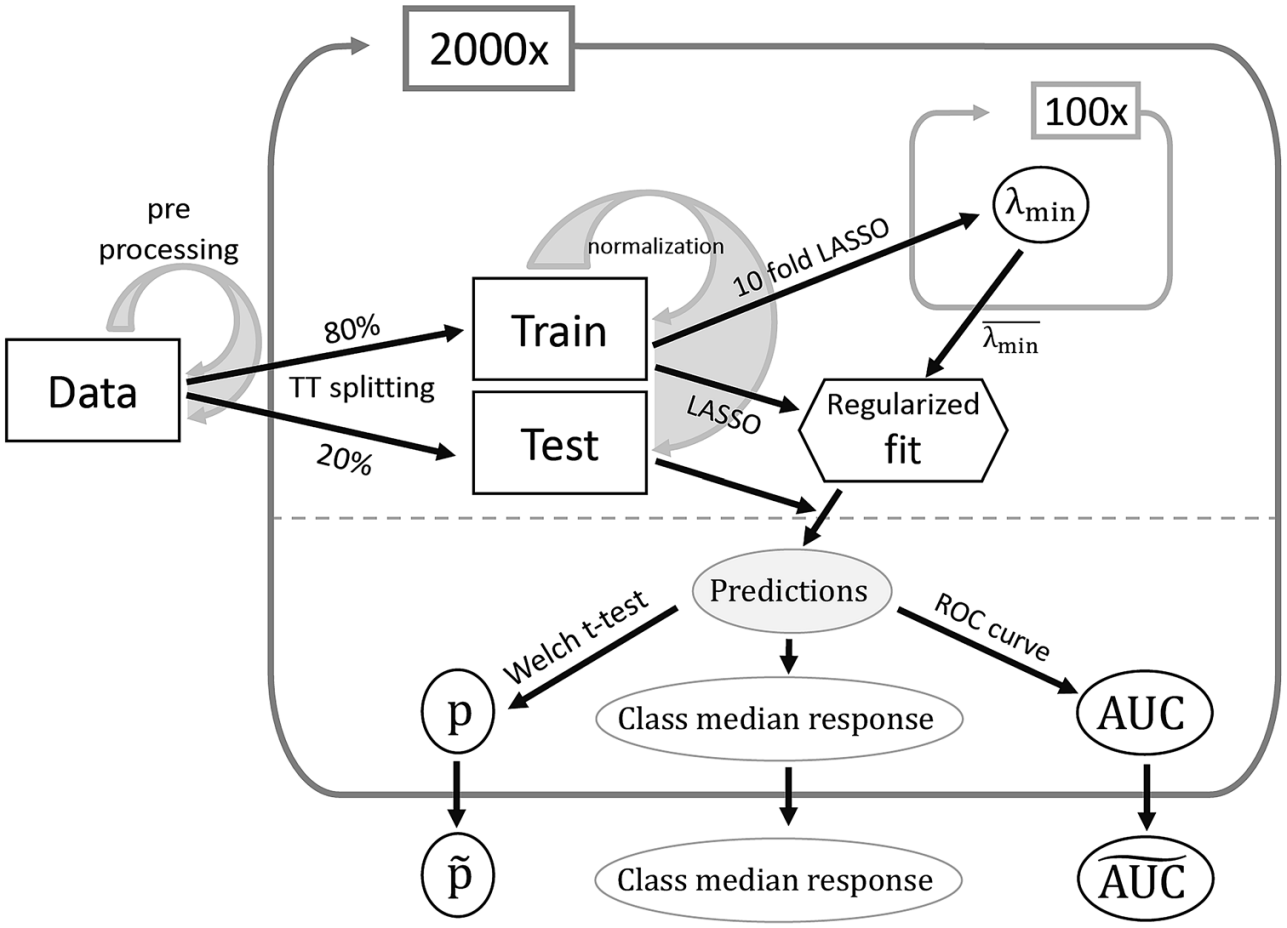
Schematics of the data analysis process. The data are pre-processed (imputation, correlated and low-variance variables removal) before training-test splitting. The normalization process (standardization) is based on the training set only but applied to both sets. The optimal value for λ (λ_min_) is found by repeating 100 times a 10-folded LASSO and by averaging the λs maximizing the AUC. The final model built on the training set with λ_min_ is then used on the test set to assess the classifier performance. The whole process, starting from the training-test split, is repeated 2000 times to provide the AUC distribution

The whole splitting-training–testing process was re-iterated 2000 times, to provide a statistically relevant performance distribution. The median p values and AUC were considered representative for the whole distribution. Median and interquartile ROC curves were computed as the median and interquartile values of the 2000 sensitivity values for each specificity bin. Refer to Fig. 1 for a scheme of the analysis process.

#### Results

The mean (range) ages of the C + , C− and CL patients were 66.5 ± 14.9 (35–100) years, 65.1 ± 19.5 (25–90) years and 61.7 ± 18.3 (36–85) years, respectively.

In the group of C + patients, 41/68 were male (60.3%), 27/68 (39.7%) were female; in the group of C- patients, 21/38 were male (55.3%), 17/38 (44.7%) were female; in the group of CL patients, 5/9 were male (55.6%), 4/9 (44.4%) were female.

On the basis of the CT findings, a CO-RADS score of 6 was assigned to all the C + positive patients. Mean and median CO-RADS score for C− patients were 2.9 and 3, whereas mean and median CO-RADS score for CL patients were 4.4 and 5.

**Fig. 2:**
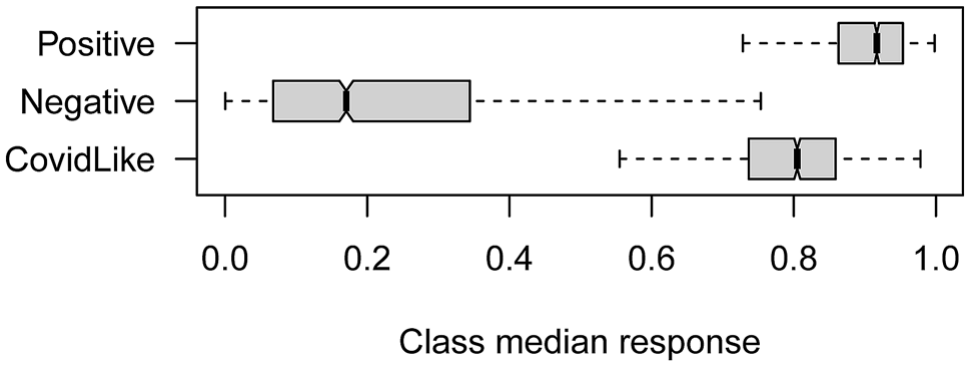
Distribution of the class median response among the 2000 data splits. Responses of 0 and 1 indicate a 100% probability of being C− and C + , respectively. The class median response of CL patients is always higher than 0.5, indicating that these cases are similar to C +

The first model was trained on C + and C− patients only. The boxplot related to the distribution of the class median response for the three groups within the 2000 repetitions is reported in Fig. 2. The median response of the CL patients (0.80) is more similar to the C + ones (0.92) than C− (0.17). Indeed, the median *p* value obtained for the Welch test comparing the C + and CL classes (p_C+CL_ = 0.12) indicated that the two distributions are not statistically different, whereas the value obtained comparing C− versus CL and C + versus C− (p_C-CL_ = 0.048 and p_C+C-_ < 0.001) indicated that the C- class is statistically separated from the other two.

**Fig. 3:**
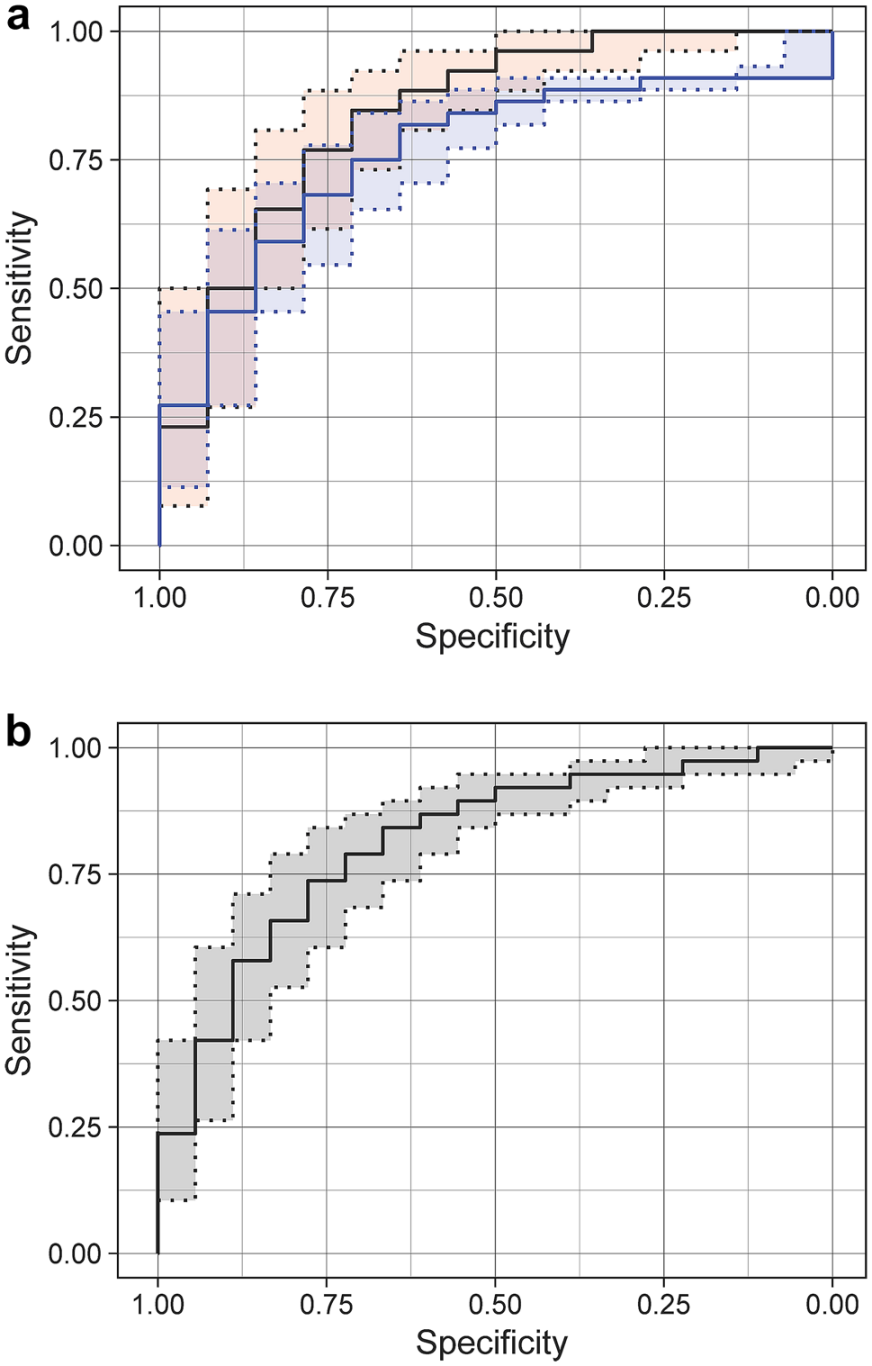
**a** Median ROC curves for the first model (trained with the exclusion of CL cases). The red curve represents the response for a Test set composed only of C + and C− patients. The blue one represents the performance with the introduction of CL in the Test set. **b** Median ROC curve for the second model, trained and tested with the inclusion of CL patients

The first model classified C + and C− pneumonias with a median AUC of 0.83 (IQR = [0.77, 0.89]). In the ROC curve corresponding to the median AUC, the optimal specificity and sensitivity are 79% and 77%, respectively, with a threshold of 0.60. This yields to a positive predictive value (PPV) of 87%. The median ROC curve for this model is reported in Fig. 3a in red.

The median ROC curve computed using the first model but merging the CL and C + cases in the test set, provided a median AUC of 0.77 (IQR = [0.71, 0.83]). The optimal threshold in this situation was 0.81, providing a specificity of 93% and a sensitivity of 57%, with a PPV of 96%. The median ROC curve in the case of inclusion of CL patients in the test set is shown in Fig. 3a in blue.

Figure 3b shows the ROC curve of the second model, where CL cases are included before the set split. In this case, the presence of CL patients does not influence the performances, providing an AUC of 0.81 (IQR = [0.75, 0.86]). The specificity and sensitivity, for the optimal threshold of 0.90, are 94% and 66%, respectively, with PPV of 96%. In this case, the ML algorithm is more likely to classify CL cases as positive than negative.

## Discussion

We built a double ML model based on whole lung radiomic features. We decided to perform whole lung analysis in order to avoid segmentation bias and to include areas of apparently normal lung parenchyma which may have subtle radiomic alterations. Two recent papers adopted this approach, respectively, to develop an automated framework to detect COVID-19 in chest CT [21] and to distinguish stable from progressive COVID-19 infection, on radiomics footprint basis [23].

The first model was split in two. In the first analysis, where CL cases were excluded from both training and test sets, the model could distinguish COVID-19 positive pneumonia from COVID-19 negative pneumonia. It performed satisfactorily, with an AUC of 0.83, a specificity and a sensitivity of 79% and 77%, respectively, and a PPV of 87%. This result is consistent with a previous work where a ROI-based machine learning model was used, yielding higher sensitivity and specificity (94.16% and 88.62%, respectively) [24]; however, that work aimed at distinguishing COVID-19 pneumonia from general pneumonia, while our investigation was exclusively focused on interstitial pneumonias. Furthermore, in that work, the validation of the results (i.e., the test set) was not present and the number of cases was limited (27 General pneumonias, 34 COVID-19), potentially biasing the performance.

Another paper investigated the role of radiomics to distinguish COVID-19 pneumonia from other interstitial pneumonia. The mixed model had an AUC of 0.959 and 0.955 in the training and test sample, respectively, and was deemed clinically useful [25]. The higher performance compared to our model was probably due to the higher number of patients in both COVID-19 and other interstitial pneumonia groups. However, a single test-train split was performed, whereas in the present work a multi-split approach was adopted in order to reduce the chance of false-discovery due to a fortunate test-train split. For example, among the 2000 splits for the first model, 9 provided an AUC of 1.00, 142 an AUC above 0.95 and 427 above 0.90. Picking one of these splits without repeating the whole process would lead to excessively high estimated performances. Furthermore, in that paper, the authors did not include CL patients.

Since in CL patients the nasopharyngeal swabs were negative, but the clinical findings were suggesting COVID-19 disease and patients were treated as positive, the model trained only on C + and C− patients was used to predict the response of CL patients. The distribution of the CL median response for the first model was statistically different from the C- response (p_C-CL_ = 0.048) but not different from C + (p_C+CL_ = 0.12). The median values of the distribution of the three classes was 0.80 for CL, 0.92 for C + and 0.17 for C−. The similar response of CL and C + patients suggests that CL cases are likely to be classified as C + by the first model and that the radiomics footprint of the two groups might be compatible. The only difference between the two groups is the fact that in C + patients the nasopharyngeal swabs are true positives whereas in CL they are false negatives. These false negatives could be due to an inappropriate technique or timing [26] when collecting the sample, possibly resulting in low viral levels on the swab, insufficient for gene amplification. However, in a patient highly suspicious for COVID-19 infection with a negative swab, the presence of a whole lung HRCT radiomic footprint similar to a patient with a positive swab test may support the diagnosis, obviating the necessity to perform a more invasive sample collection, such as bronchial alveolar lavage.

The second analysis of the first model, performed including CL exclusively in the test set, reported an AUC of 0.77. This substantial drop of the performance may indicate that, even though HRCT of CL patients are morphologically similar to C + ones, there is an appreciable difference in the quantitative analysis. This may be due to a different involvement in lung parenchyma in CL patients compared to C + ones, which may reflect a lower viral load in CL patients. Recently, Zhao et al. investigated the relationships between viral load and CT findings. They found that the low viral load was negatively associated with an uneasily differentiated lesion margin, reflecting a different morphology of COVID-19 CT alteration depending on viral load [27]. This hypothesis, together with the timing of the swab, may be the reason of the false negative RT-PCR [28] result and may explain the higher sensitivity of the HRCT compared to RT-PCR [5].

The second model was built including CL cases in the training set. The overall performances of the model remained almost unchanged, with a slight decrease of 0.02 in the AUC. Even though this decrease is not statistically significant, it must be pointed out that only two CL patients per fold were included in the test set.

This study has limitations. Firstly, it is a retrospective, single-center study; a prospective, multicentric study is required to validate its findings. Furthermore, the low number of CL patients limited the potential of the classification method to guide patient management in case of negative swab test.

## Conclusion

A machine learning model based on whole lung HRCT radiomics footprint may be useful to classify COVID-19 from non-COVID-19 interstitial pneumonia. Furthermore, COVID-like patients (HRCT findings suggestive of COVID-19 with negative RT-PCR) appear to have radiomics footprints very similar to COVID-19 positive patients. This may help in the correct management of patients with clinical and radiological stigmata of COVID-19, however presenting with negative RT-PCR.

## Data Availability

Available at the authors’ institution.
